# The influence of statistical properties of Fourier coefficients on random Gaussian surfaces

**DOI:** 10.1038/s41598-017-02135-y

**Published:** 2017-05-16

**Authors:** C. P. de Castro, M. Luković, R. F. S. Andrade, H. J. Herrmann

**Affiliations:** 1 Instituto de Física, Universidade Federal da Bahia, Campus Universitário da Federação, Salvador, BA 40170-115 Brazil; 2 Computational Physics for Engineering Materials, IfB, ETH Zurich, Wolfgang-Pauli-Strasse 27, CH-8093 Zurich, Switzerland; 3 Departamento de Física, Universidade Federal do Ceará, Fortaleza, Ceará 60451-970 Brazil

## Abstract

Many examples of natural systems can be described by random Gaussian surfaces. Much can be learned by analyzing the Fourier expansion of the surfaces, from which it is possible to determine the corresponding Hurst exponent and consequently establish the presence of scale invariance. We show that this symmetry is not affected by the distribution of the modulus of the Fourier coefficients. Furthermore, we investigate the role of the Fourier phases of random surfaces. In particular, we show how the surface is affected by a non-uniform distribution of phases.

## Introduction

Two-dimensional random surfaces can be considered as a generalization of one-dimensional stochastic processes. Often, properties of natural systems, such as sea surface temperatures, rough graphene surfaces and 2D turbulence can be mapped onto random surfaces^1–6^. Their scaling properties can be characterized by a single parameter known as the Hurst exponent, *H*. This exponent is related to the degree of spatial correlation between two points on the surface. For all *H* > −1 the surfaces are long-range correlated, rough and self-affine^6–8^. Uncorrelated surfaces correspond to an *H*-value of −1.

Much can be learned about the properties of random surfaces by studying the paths of constant height (lines) extracted from them^7, 9–13^. Empirical and numerical studies of these paths suggest that, at the height corresponding to the percolation threshold, they are scale invariant and their fractal dimension depends on the Hurst exponent *H* ^10, 14, 15^. In some cases they also have an additional symmetry reflected by the conformal invariance of these paths^4, 6^. This means that the statistics of such curves is covariant with respect to local scale transformations^16^.

There exist several methods to generate random surfaces^17^. In this work, we consider the Fourier Filtering Method (FFM), where one first creates a random surface in the reciprocal space and then Fourier transforms it into real space. Our results are based on high performance calculations, large system sizes as well as tens of thousands of samples.

In the context of random surfaces, it is taken for granted that critical exponents, such as the fractal dimension of the percolation cluster and its perimeters, or those related to the correlation length and the susceptibility, depend only on *H* ^18, 19^. In the case of conformal invariance, the current view is not as straightforward. Although conformal theory is not the main topic of this paper, it serves as motivation for our investigations^20–24^. In particular, it is worth noting that random curves with well defined Hurst exponents do not necessarily exhibit conformal invariance. For example, Bernard *et al*. observed conformal invariance in the iso-height lines of vorticity fields of 2D turbulence^4^. They also showed, however, that this property is violated for iso-height lines extracted from surfaces with the same Hurst exponent but with randomly distributed phases of the surface heights in Fourier space. Therefore, it seems that it is not only the Hurst exponent that plays a determinant role in conformal invariance. Furthermore, outside the context of conformal invariance, Kalda, J. shows that gradient-limited surfaces are not characterized only by *H* ^19^.

The possible dependence of conformal invariance on phase correlations^4^ and the existence of scale-invariant curves with scale-dependent critical exponents^19^, has therefore motivated us to investigate whether the scale invariance of iso-height lines of random Gaussian surfaces is also affected in a similar way.

Given that each point of the random surface in reciprocal space is determined by the phase, as well as the magnitude of a complex number, for the sake of completeness we also study the effects of the latter on the scale invariance of the iso-height lines. Therefore, we investigate how the critical exponents, some of them defined by Kodev, J. and Henley, C. L.^15^, are influenced by Fourier phases, especially their correlations, as well as the distribution of the magnitudes of the Fourier components.

We show that a non-uniform distribution of Fourier phases introduces symmetries in random surfaces and that a change in the phase correlation length in Fourier space causes a translation of the surface in real space. Furthermore, our results show that changes in the shape of the distribution of Fourier magnitudes, without altering their correlations, have the sole effect of modifying the height magnitudes of the random surfaces. None of the variations described above change significantly the H-dependence of the critical exponents as conjectured by Schrenk K. J. *et al*. in ref. 10.

## Method

### Random gaussian surfaces

A set of random real numbers may be interpreted as a surface, where each number corresponds to the height *h*(**x**) = *h*(*x*_1_, *x*_2_) at coordinate *x*_1_ and *x*_2_^10, 12, 13, 17, 25^. In order to create correlated random surfaces, we used the Fourier Filtering Method (FFM)^26–31^, which consists in defining a complex function *η*(**q**) in Fourier space and then taking the inverse transform to obtain *h*(**x**). The complex Fourier coefficients *η*(**q**) can be written in the form

1 $$\eta ({\bf{q}})={\mathtt{c}}({\bf{q}})\,\exp (2\pi \varphi ({\bf{q}})),$$

where **q** = (*q*_1_, *q*_2_) is the frequency in Fourier space, ${\mathtt{c}}({\bf{q}})$ the magnitude and *ϕ*(**q**) the phase. In order to obtain a random surface with the desired properties, we chose the power spectrum *S*(**q**) of the surface in the form of a power law such that

2 $$S({\bf{q}})\sim {|{\bf{q}}|}^{-{\beta }_{c}}={(\sqrt{{q}_{1}^{2}+{q}_{2}^{2}})}^{-{\beta }_{c}}$$

where *β*_*c*_ = 2(*H* + 1)^17^ defines the Hurst exponent. Then, we apply the power-law filter to a real random variable *u*(**q**) obtaining for the magnitude

3 $${\mathtt{c}}({\bf{q}})={[S({\bf{q}})]}^{\mathrm{1/2}}u({\bf{q}}).$$

In general, *u*(**q**) is Gaussian distributed with finite variance, $\varphi ({\bf{q}})\in [0,1]$ is uniformly distributed noise and ${\mathtt{c}}({\bf{q}})$ must satisfy the conjugate symmetry condition, ${\mathtt{c}}(\,-\,{\bf{q}})=\overline{{\mathtt{c}}({\bf{q}})}$ ^17^. Without loss of generality, we will consider the case where *u*(**q**) has unit variance.

The choice of the power spectrum as a filter is justified by the Wiener-Khintchine theorem^17, 32^, which states that the autocorrelation function, *C*(**r**), of a time series is the Fourier transform of its power spectrum. Therefore, from the inverse discrete Fourier transform of *η*(**q**) we obtain *h*(*x*_1_, *x*_2_)

4 $$h({x}_{1},{x}_{2})=\sum _{{q}_{1}=0}^{N-1}\sum _{{q}_{2}=0}^{N-1}\,\eta ({q}_{1}{q}_{2})\,\exp (\,-\,2i\pi ({q}_{1}{x}_{1}+{q}_{2}{x}_{2}))$$

with the desired power-law correlation function^7, 10, 17^

5 $$C({\bf{r}})\sim {{\bf{r}}}^{2H}.$$

According to the definition above, if *H* = −1 and therefore *β*_*c*_ = 0, the power spectrum in eq. 2 becomes independent of the frequency, giving rise to uncorrelated surfaces. As *H* increases from −1, height-height correlations are introduced into the surface.

For any random surface defined on a lattice with *H* ≥ −1, the percolation threshold *p*_*c*_ can be determined using the well established *rank method*. Moreover, a conjecture was recently put forward for the *H*-dependence of the fractal dimension and critical exponents at the corresponding critical point *p*_*c*_^10^. It should also be noted that as a consequence of the extended Harris criterion^11, 18, 33–36^, there are going to be some critical exponents of 2D systems that are not influenced by correlation effects related to $H\in [\,-\,1,-3/4]$, implying that for those Hurst values, the exponents are expected to be the same as for the uncorrelated system^10^.

In the case of self-affine surfaces, for which *H* > 0, the percolation threshold is not well defined, since there is no unique value of the surface height at which the system percolates. Nevertheless, in this case also, it is possible to extend some concepts of percolation theory and relate them to *H* ^7, 8^.

### Clusters and perimeters

**Figure 1:**
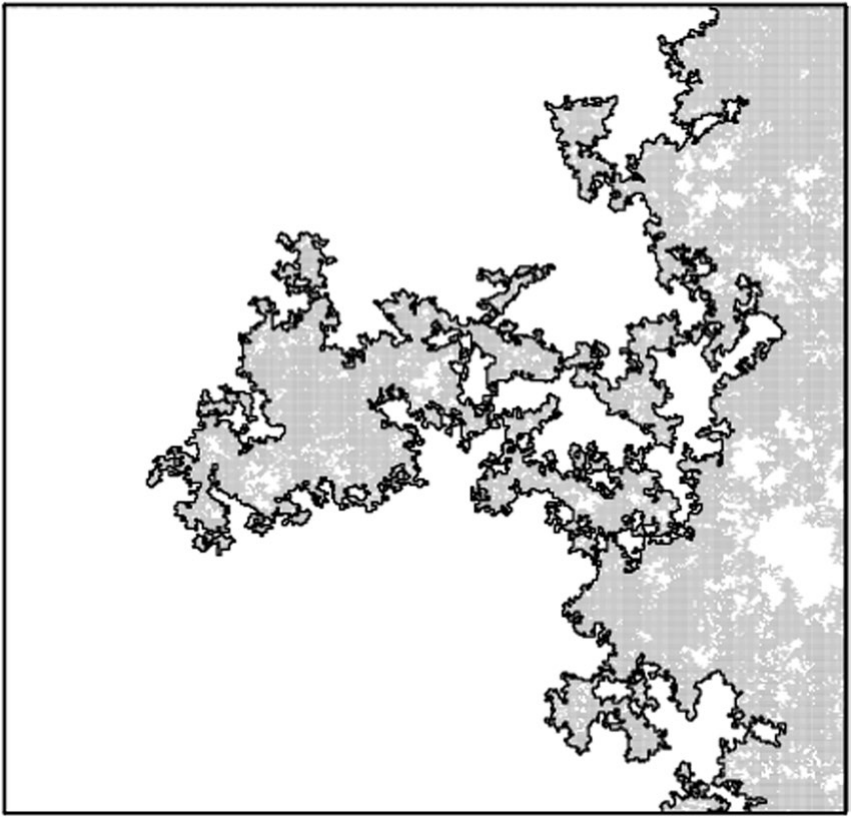
Schematic picture of the percolating cluster (light gray) connecting the top of the square with the bottom. The white region corresponds to sites that do not belong to the percolating cluster (unoccupied sites and other clusters) and the black line is the external (complete) perimeter. This map was generated by OriginPro 2016 (64-bit) Sr1 b9.3.1.273 (http://www.originlab.com/).

After generating the discrete random Gaussian surfaces, we use the rank method^37^ to reach the percolation threshold in the following way. One first ranks all sites in the landscapes according to their height, from the smallest to the largest value. Then, a ranked surface is constructed where each site has a number corresponding to its position in the rank, so that the following percolation model can then be defined. Initially, all sites of the ranked surface are unoccupied. Then the sites are occupied one by one, according to the ranking. At each step, the fraction of occupied sites *p* increases by the inverse of the total number of sites on the surface, thereby changing the configuration of occupied sites. By continuing this procedure a critical height *h*_*c*_ is reached at which the occupied neighboring sites create a spanning cluster (percolation cluster) that connects two opposite borders of the surface (Fig. 1). At the critical height, the fraction of occupied sites reaches the percolation threshold *p*_*c*_. From the percolation cluster we extracted the fractal iso-height lines that correspond to the *complete perimeter* and *accessible perimeters* ^7, 10, 38, 39^. The complete perimeter consists of all bonds between the percolating cluster and unoccupied sites. This is illustrated in Fig. 1, where light-gray represents the percolating cluster and the black line follows the complete perimeter.

The accessible perimeter is obtained by eliminating from the complete perimeter all line segments within fjords with a bottleneck equal to the length *r* of the current stick. This procedure stems from the yardstick method used to measure the perimeter’s fractal dimension. Here, for each value of *r*, the length of any curve is defined by the number of straight yardsticks *N*_*r*_ required to go from one extreme to the other by jumping from one point on the curve to the next at a distance *r*. Then, the fractal dimension *df*_*p*_ is defined by

6 $${N}_{r}\sim {r}^{-d{f}_{p}}.$$

**Figure 2:**
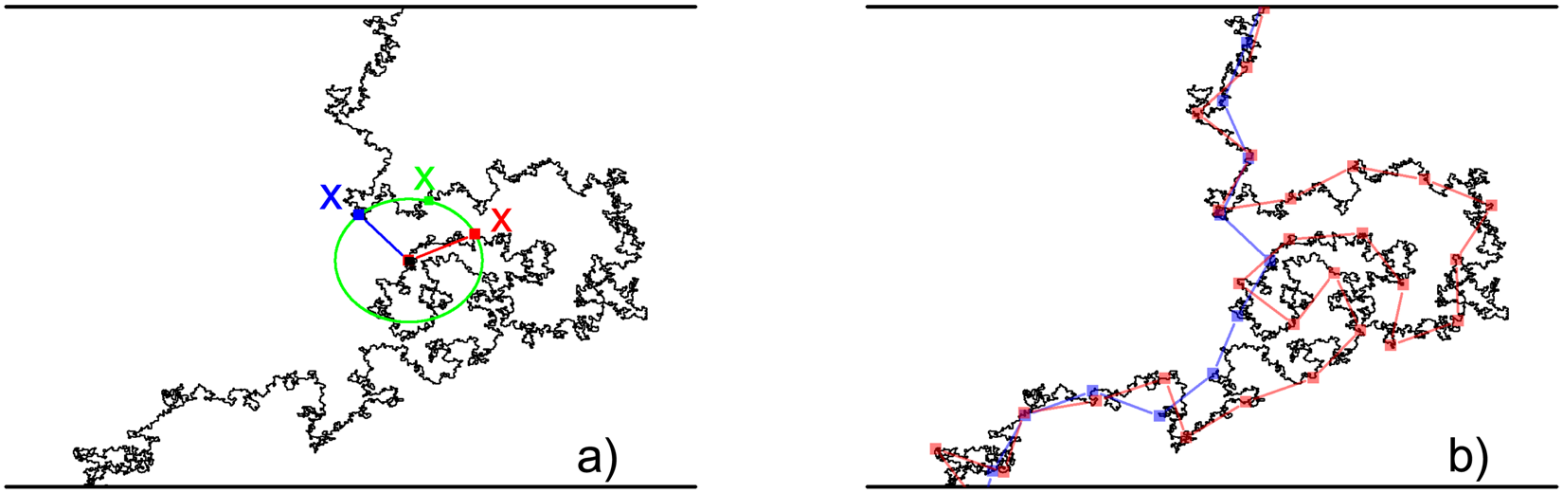
(**a**) Illustration of the rules used to compute the fractal dimension of the complete and accessible perimeters with the yardstick method. Suppose the sticks start to follow the coast from the bottom. The green circle shows the region of coast accessible to a particular stick. The **X**’s represent the next possible starting points of that particular stick. If the closest point along the coast (red **X**) is always chosen as the next starting point, we obtain the complete perimeter. If, on the other hand, the most distant point (blue **X**) is chosen, then we obtain the accessible perimeter. Here, points such as the green X, which are neither the closest nor the most distant from the center of the circle, are disregarded. (**b**) Paths made by sticks of equal sizes of the complete (blue sticks) and accessible (red sticks) perimeters. These maps were generated by OriginPro 2016 (64-bit) Sr1 b9.3.1.273 (http://www.originlab.com/).

Figure 2a shows an arbitrary curve where the black dot, in the center of the green circle, indicates the current stick position. During this specific search for the next point on the curve, three possible positions indicated by red, green and blue X’s are found. If the option to always take the closest position along the curve (red X) is made, the complete perimeter is obtained. On the other hand, if one always takes the most distant point along the curve (blue dot), which does not avoid the external border, the accessible perimeter is obtained. Indeed, this rule skips points inside fjords and accesses only the external boundary of the coast, independently of the chosen direction (in the case of Fig. 2 either bottom-up or top-down), because there is no preferential correlation direction. Figure 2b shows the difference between the considered paths for one particular stick size.

## Results and Discussion

Having described the method for generating random surfaces using two sets of random variables, *u*(**q**) and *ϕ*(**q**), we now discuss how a surface is affected by changing the form of their respective distributions.

**Figure 3:**
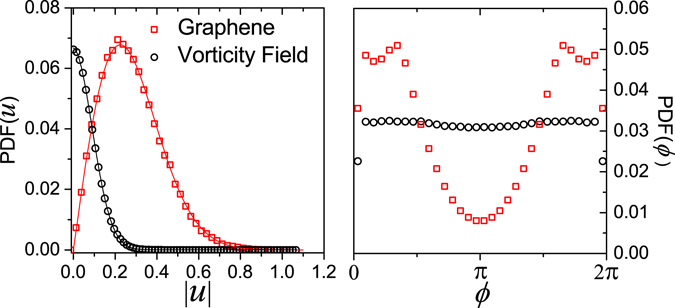
Probability density function of |*u*(**q**)| (left) and *ϕ*(**q**) (right) in the case of the graphene sheet (red squares) and the vorticity field (black circles). The red and black curves in left panel are best fits for $f(|u|)\propto {c}_{1}|u|\,{\exp }^{-{c}_{2}{|u|}^{2}}$ and the Gaussian function, respectively.

Although common^17^, it is not always the case that *u*(**q**) follows a Gaussian distribution and *ϕ*(**q**) a uniform one. For example, Giordanelli *et al*.^6^ found that for graphene sheets *u*(**q**) is well fitted by $f(|u|)\propto {c}_{1}|u|\,{\exp }^{-{c}_{2}{|u|}^{2}}$, where *c*_1_, *c*_2_ parameters of the fit. They also found that the Fourier phase distribution *ϕ*(**q**) is bi-modal rather them uniform^6^, as ilustrated in Fig. 3. On the other hand, for the vorticity field of 2D turbulence, we found through independent analysis that *u*(**q**) follows a Gaussian distribution and that *ϕ*(**q**) is nearly uniformly distributed used. Both *u*(**q**) and *ϕ*(**q**) are obtained by creating a histogram of the random terms of the Fourier transform of the surface being studied.

By an adequate choice of the u(q) and *ϕ*(*q*) distributions, we were able to generate FFM surfaces that are statistically similar to those in graphene and the vorticity fields in 2D turbulence. This allowed us to investigate how different distributions influence the resulting random surfaces.

### Fourier phases

**Figure 4:**
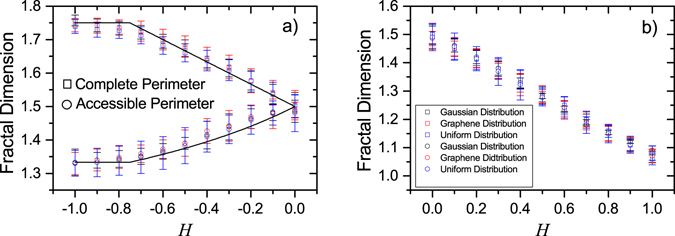
Fractal dimension of the complete and accessible perimeters as a function of *H*, for (**a**) *H* < 0 and (**b**) *H* > 0, and different *ϕ*(**q**) distributions. In (**a**), the black lines are conjectures proposed by Schrenk K. J. *et al*.^10^. All values are averages over at least 10^4^ samples and error bars are defined by the variance of the distribution.

We start by showing the results obtained for three different *ϕ*(**q**) distributions (Gaussian, uniform, and the one found by Giordanelli *et al*. in graphene), while always keeping the same Gaussian distributed *u*(**q**). Applying the method described in the previous section, we obtained the dependence of the fractal dimension of the complete (${d}_{f,H}^{com}$) and accessible (${d}_{f,H}^{acc}$) perimeters on *H*, as illustrated in Fig. 4. Since exact values for the fractal dimension of those perimeters are known only for *H* = −1 and *H* = 0, all other proposed analytical dependencies on *H* are conjectures supported by numerical results^10, 40–42^. In the case of uncorrelated surfaces, ${d}_{f,H=-1}^{com}=7/4$ and ${d}_{f,H=-1}^{acc}=13/10$. When *H* increases from −1, the fractal dimension of complete and accessible perimeters start to converge. Once the surfaces are described by a discrete Gaussian Free Field^43^ for *H* = 0, the results consistently indicates $d{f}_{H=0}^{com}=d{f}_{H=0}^{acc}=3/2$. Our results therefore point towards the absence of any dependence of $d{f}_{H}^{com}$ and $d{f}_{H}^{acc}$ on the shape of the distribution of *ϕ*(**q**). As shown in Fig. 4, the *H*-dependence of $d{f}_{H}^{com}$ and $d{f}_{H}^{acc}$ agrees with the conjectures by Schrenk K. J.^10^ for both, long-range correlated (Fig. 4a) and rough surfaces (Fig. 4b).

**Figure 5:**
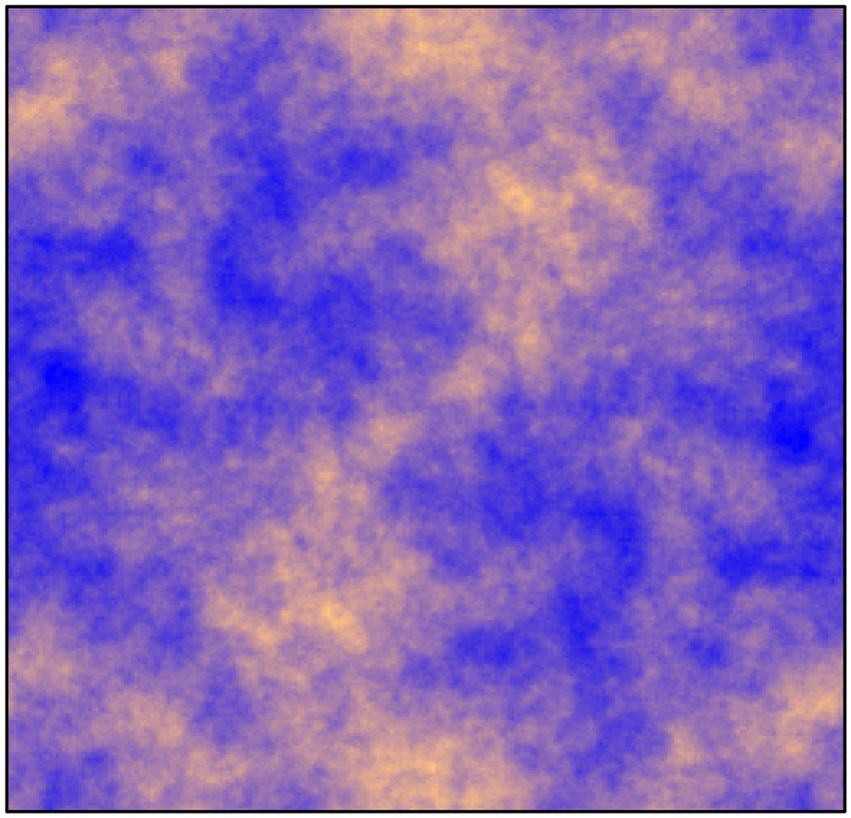
Surface map with inversion symmetry with respect to the center. This symmetry of the surface results from the use of a Gaussian distribution *ϕ*(**q**) with a small variance *σ* = 0.001.

At first glance, the influence of the Fourier phases on the random surface might not be obvious. However, we notice that the phase distribution mainly influences inversion symmetries with respect to the center of the surface, as shown in Fig. 5. In order to introduce this effect, *ϕ*(**q**) is given by a Gaussian distribution with a very small variance, i.e. a peaked distribution. In Fig. 5 it is possible to identify the same morphological structures when the figure is rotated by an angle *π*. This occurs because, as the variance goes to zero, the distribution values of the Fourier phases converge to a constant (the mean value) accentuating the symmetry effect^44^. In the limiting case of zero variance the distribution collapses onto a constant value, producing a perfect inversion symmetry in real space. Therefore, in order to avoid this symmetry effect a uniform distribution is used.

### Correlated phases

**Figure 6:**
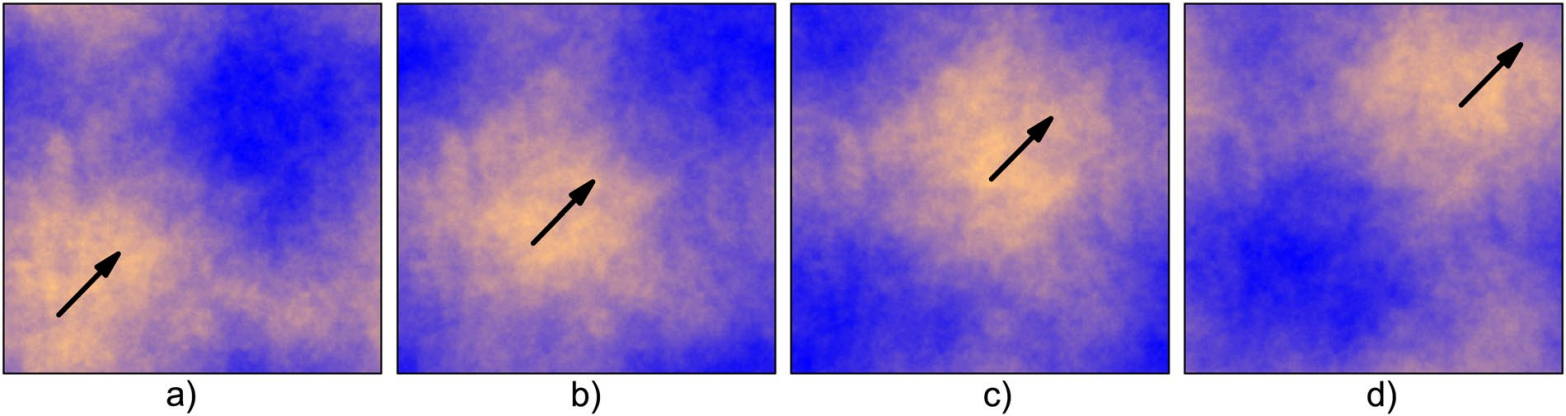
Maps of phase correlated surfaces. Panels (a–d) show examples of surfaces with *H* = 0.5 and *H*_*phase*_ = −0.9, −0.2, 0.1, and 0.4 respectively. The arrows serve as a guide to show the linear translation of the random surface due to correlations introduced between the Fourier phases.

It turns out that the Fourier phases from the vorticity fields and graphene sheets that we analyzed are uncorrelated^6^. Nevertheless, in order to understand how correlations affect random surfaces, we generated some samples with artificially correlated Fourier phases. For this purpose, we introduced correlations in the Fourier phases by applying the FFM twice. First we used the FFM to create a surface of correlated random phases in **q**-space with Hurst exponent *H*_*φ*_, according to the eq. 1. Applying the FFM again, we generate Gaussian surfaces with Hurst exponent *H* and with the desired coefficients and correlated Fourier phases. Using always the same distributions of *ϕ*(**q**) and *u*(**q**) and keeping fixed the value of *H* and the seed of the random number generator, we studied the changes in the surface caused by a change in *H*_*φ*_. We found that the correlation of Fourier phases causes a linear translation of the random surfaces (Fig. 6). A change in *H*_*φ*_ modifies the slope of the power spectrum (eq. 2), causing all sites of *ϕ*(**q**) to shift proportionally, which means that a constant value is added to the Fourier phases, causing a translation on the random surface (in real space)^44^.

### Magnitude of the Fourier coefficients

**Figure 7:**
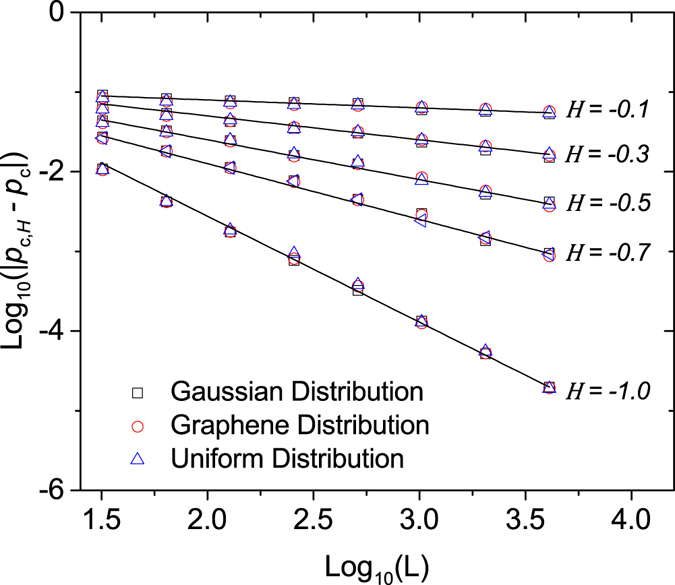
Scale analysis of the convergence of the percolation threshold *p*_*c*,*H*_. For the square lattice, the site percolation threshold *p*_*c*_ for uncorrelated surfaces is ${p}_{c}\simeq 0.592746$. The black lines serve as guides to the eye with slope *H* = −1/*ν*_*H*_^10, 38, 39^.

We generated sets of random surfaces, each having *ϕ*(**q**) uniformly distributed but with a different distribution of *u*(**q**): Gaussian, uniform and the distribution found by Giordanelli *et al*. in graphene. We then determined the average values of two critical exponents of percolation corresponding to each set of surfaces with a different *u*-distributions. We first considered the *H*-dependence of the correlation length critical exponent *ν*_*H*_ for −1 ≤ *H* ≤ 0. It is well established that the critical point ${p}_{c}\simeq 0.592746$ ^11, 13, 18, 35^ is the infinite system size limit of the percolation threshold *p*_*c*_(*H*, *L*), which is *H*-dependent for finite system sizes, *L*. Furthermore, the expected scaling behavior^10, 38, 39^ is

7 $$|{p}_{c}(H,L)-{p}_{c}|\sim {L}^{-\mathrm{1/}{\nu }_{H}},$$

with *ν*_*H*_ = −1/*H* ^10, 45, 46^. Our numerical results in Fig. 7 not only confirm that the scaling relation in eq. 7 is respected no matter which one of the three *u*-distributions we use but also that the value of *p*_*c*_ remains unchanged.

A consequence of the scaling relation in eq. 7 is that in the asymptotic limit it is sufficient to compute the critical exponents at the percolation threshold, *p*_*c*_.

At this critical point, the percolation cluster is a fractal with fractal dimension *d*_*f*_. The occupancy, *M*_*max*_, which is the number of sites that belong to the percolation cluster, scales with lattice size *L* as,

8 $${M}_{max}\sim {L}^{{d}_{f}}.$$

**Figure 8:**
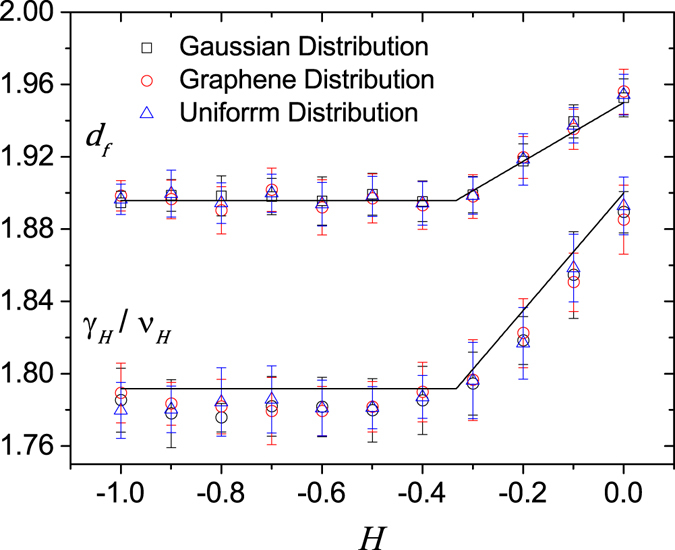
Fractal dimension *d*_*f*_ of the percolation cluster and critical exponent ratio *γ*_*H*_/*ν*_*H*_ as a function of the Hurst exponent *H* for surfaces with different distributions of *u*(**q**). The black lines are conjectures proposed by Schrenk K. J. *et al*.^10^ based on the hyperscaling relation^18^. All values are averages over at least 10^4^ realizations and error bars are defined as the variance of the distribution of their values.

Using eq. 8 we recovered numerically the value of the fractal dimension as a function of the Hurst exponent. In Fig. 8 our results show that the value of the fractal dimension of the percolation cluster remains the same for all three distributions of *u*(**q**).

We also checked the *H*-dependence of the susceptibility critical exponent *γ* by considering the scaling behavior of *m*_2_, the second moment of the distribution of the cluster sizes at *p*_*c*_ defined as^18^

9 $${m}_{2}=\sum _{k}\,\frac{{M}_{k}^{2}}{N}-\frac{{M}_{max}^{2}}{N}.$$

Here, the sum runs over all clusters, where *M*_*k*_ is the mass of cluster *k*, and we use the fact that the following scaling behavior holds^18^:

10 $${m}_{2}\sim {L}^{{\gamma }_{H}/{\nu }_{H}}.$$

For uncorrelated percolation (*H* = −1), *γ*_*H*=−1_ = 43/18, *ν*_*H*=−1_ = 4/3, so that *d*_*f*_ = 91/48 and *γ*_*H*=−1_/*ν*_*H*=−1_ = 43/24^18^. Figure 8 shows the dependence on $H\in [\,-\,1,0]$ of both critical exponents, the fractal dimension of the percolation cluster and the exponent ratio *γ*/*ν*, for different distributions of *u*(**q**).

In conclusion, our results suggest that both exponents, *d*_*f*_ and *γ*/*ν*, are independent of the distribution of *u*(**q**).

## Conclusions

We considered two concrete examples of random surfaces, namely, the vorticity field of turbulent systems in two dimensions and rough graphene sheets. We investigated how these random surfaces and in particular the critical exponents are influenced by the presence of phase correlations and by changes in the distribution of the Fourier coefficient magnitudes and Fourier phases. Our results show that the Fourier phases distribution of the vorticity field and graphene sheets, within error bars, lead to the same value for the fractal dimension of the complete and accessible perimeters. We also showed that long-range phase correlations in Fourier space lead to a translation of the random surfaces, and that they do not have any influence on their statistical properties. For different distributions of magnitude of Fourier coefficients our results suggest there is no *H* dependence of the fractal dimension of the percolation cluster and susceptibility exponent. In addition, we recovered for the critical exponents the same *H*-dependence as conjectured by Schrenk K. J.^10^. Although we have only considered three examples of Fourier coefficient distributions, we do not expect different results for any other distribution with finite variance.
